# Quality of work community and workers’ intention to retire

**DOI:** 10.1007/s00420-021-01826-4

**Published:** 2022-01-07

**Authors:** Subas Neupane, Saila Kyrönlahti, Hanna Kosonen, K. C. Prakash, Anna Siukola, Kirsi Lumme-Sandt, Pirjo Nikander, Clas-Håkan Nygård

**Affiliations:** 1 Unit of Health Sciences, Faculty of Social Sciences, Tampere University, Tampere, Finland; 2 Gerontology Research Center, Tampere University, Tampere, Finland; 3 Department of Public Health, University of Turku and Turku University Hospital, Turku, Finland; 4 Clinical Medicine, Faculty of Medicine and Health Technology, Tampere University, Tampere, Finland

**Keywords:** Work environment, Psychosocial factors, Older workers, Postal service, Retirement intention

## Abstract

**Purpose:**

To study the workers’ perception of the quality of work community and its association with intention to retire early, separately among women and men working in Finnish postal service.

**Methods:**

A questionnaire survey was sent to all Finnish postal services employees aged ≥ 50 years in 2016 and 44% (*n* = 2096) replied to the survey (mean age 56.3, 40% women). Employee’s intention to retire before statutory retirement was measured on a scale of 1–5 and dichotomized. The quality of work community was defined by four composite variables: equality at work, flexibility at work, supportive work environment and health or other reason and trichotomized by their tercile values. Odds ratio (ORs) and their 95% confidence intervals (CIs) for associations of quality of work community with intention to retire were calculated separately for men and women using log binomial regression models adjusted for potential confounders.

**Results:**

About one-third of respondents intended to retire early with no significant gender difference in retirement intention. Low equality at work (women OR 2.77, 95% CI 1.60–4.81; men 2.84, 1.80–4.48) and low flexibility at work (women 3.30, 1.94–5.60; men 2.91, 1.88–4.50) was associated with higher likelihood of intention to retire. Among women intention to retire was found less likely due to low supportive work environment (0.52, 0.31–0.89) and among men due to intermediate health or other reason (0.65, 043–0.98).

**Conclusion:**

The results highlight the importance of the quality of work community as well as the promotion of work-related health in order to encourage employees to remain at workforce for longer.

## Introduction

The world is facing a challenge of population aging and parallelly, labor force is shrinking (Aiyar and Ebeke 2016). Owing to low fertility and increasing life expectancy, the share of population at working age (20–64 years) will drop. This means a challenge for policy makers to find ways to keep people working longer to balance the dependency ratio (European Commission 2021). It is estimated that the median age of the labor force will increase from 41.4 years in 2017 and reach 42.6 years in Europe by 2030 (Kühn et al. 2018), which means an increased dependency ratio in the future. This suggests that people will work additional years to compensate for the decreasing share of working age population (OECD 2019). The current COVID-19 crisis has added turbulence to the labor markets by increasing the unemployment rate and created further economic impacts in many countries (OECD 2020). This may be a temporary problem but may also cause long-term consequences on labor markets.

Organization for Economic Cooperation and Development (OECD) have echoed the call to postpone retirement and create stronger incentives for workers to remain longer in working life. As a response, many European countries have already introduced policies in order to encourage their workforce to make voluntary decisions of prolonging working careers beyond statutory pensionable age (Wahrendorf et al. 2013). Financial advantage for those working beyond pensionable age was introduced in Finland in 2005 through a pension reform, which motivated ageing workers to prolong their working careers. Especially public sector employees can retire on a statutory basis after age of 63 but at the latest before the age of 68 years. Later, in 2017 through an extensive pension reform, Finland introduced a standardized provision on determination of pensions at different ages and 3 months annual rise on retirement date thereafter (Eläketurvakeskus 2017). However, there was an exception for some public sector employees who chose to keep their earlier retirement age (e.g. 58 years for practical nurses and 60 years for primary school teachers). Based on the previous findings from multiple countries, retirement tends to bring relief for those who suffered from poor self-rated health, sleep disturbances, fatigue, musculoskeletal disease, depression or headaches (Westerlund et al. 2009; Stenholm et al. 2016; Neupane et al. 2018). Therefore, the retirement process cannot be the same for all individuals. In addition to health-related factors, earlier research highlights the role of physical work environment, job satisfaction (Nilsson 2020; van der Zwaan et al. 2019), psychosocial work environment (Thorsen et al. 2016a, b, c; Carr et al. 2016), financial benefits (Sewdas et al. 2017), overall satisfaction to working life and work ability (Prakash et al. 2019) on workers’ intention to continue working beyond retirement age. On the other hand, stressful work environment has been reported as a major barrier to prolong working careers beyond statutory retirement age (Carlstedt et al. 2018; Carr et al. 2016; Hintsa et al. 2015). Organizational changes, conflicts at work, significant work pressures, high physical work demands and lacking opportunities to utilize one’s skills and knowledge at work within the organization have also been reported as factors that push people towards retirement (Reeuwijk et al. 2013).

The retirement decision is complex and needs to be seen from multiple perspectives as it involves multiple factors and aspects of life. Studies have highlighted the role of family, life partners or close friends (Nilsson et al. 2011) and work–life balance (Meng et al. 2020) on the retirement decision. Factors outside of work such as enjoying life, having increased flexibility, more time to spend with a spouse and grandchildren, and taking care of others, have also been studied in relation to retirement intentions (Reeuwijk et al. 2013). Using a cross-sectional design, we aimed to study how the quality of work environment is associated with intention to retire early before the statutory retirement age, separately among women and men, working in Finnish postal service. We also studied the interaction effect of quality of work environment and gender with respect to the intention to retire early.

## Methods

A national-level survey among Finnish postal services employees was conducted in 2016. The Finnish Postal service is one of the biggest public sector employers in Finland, employing more than 20,000 employees in 2017. A questionnaire was sent to all workers aged ≥ 50 years in the year 2016 and 44% (*n* = 2096) replied to the survey. This study utilized data from 1965 subjects who had complete information on major demographic and work-related variables. The mean age of the study population was 56.30 years (standard deviation 3.43) and 40% were women.

The study was approved by the Academic Ethics Committee of Tampere Region (ethical approval number: 32/2016).

### Measurement of variables

#### Intention to retire

The outcome variable, ‘intention to retire’ was measured on a scale of 1–5 at follow-up with a question “Have you considered that you could retire due to health or other reasons before your official retirement age?” The response options were 1 = I have not thought about this, 2 = I have thought sometimes, 3 = I have thought often, 4 = I have already filled application for a pension, 5 = I do not know. Intention to retire was defined if the participants responded 3–5 (Stynen et al. 2016).

### Quality of work community

#### Reasons to continue working beyond lowest pensionable age

To investigate the reasons that influence respondents’ decision to retire the participants were asked a question: “What kind of things would make you continue to participate in working life after the lowest pensionable age?” The answer options included 10 items, from which the respondent could choose up to three options (‘good and supportive work community’, ‘good and functional working environment’, ‘financial aspects’, ‘meaningful, interesting and challenging work’, ‘lightening of the work’ ‘work time flexibility’, ‘my health’, ‘other reason’, ‘I would not continue for any reason’ and ‘I do not know’). Each item was measured as yes/no. The information from the first eight of these initial items was comprised using principal component analysis (PCA). PCA was used to reduce the dimensionality of data by transforming the number of variables into a smaller set which represents the larger set. The new variables that are defined from PCA are linear functions of the original variables from the larger set (Jolliffe and Cadima 2016). In our analysis, PCA showed that 46% of the variation in the original items could be explained by three components. The components were named supportive work environment, flexibility at work and health and other reasons. The transformed continuous variables were then each categorized into three equal categories by using tercile values of the standardized factor variables as cutoff points.

#### Equality at work

Equality at work was assessed with five questions in the questionnaire, each measured on a scale of 0–10 (0 = not at all, 10 = very much). The factor structure of these variables (‘feeling of the appreciation at workplace’, ‘confidence towards the employer’, ‘commitment to the work’, ‘motivation’ and ‘treated fairly at workplace’) was studied with PCA which showed that an one-factor solution best fitted the data. This factor explained 62% of the variation in the original items and was named as Equality at work. The transformed variable was trichotomized using tercile values of the standardized factor score as cutoff points. Accordingly, the final variable describing equality at work had three equal categories: low, intermediate and high.

### Covariates

Participants reported their working hours as regular day work, regular two-shift work or night or other working hours. Number of diseases was defined as the self-reported number of physicians diagnosed disease and was categorised as 0 vs 1, if the participants had one or more of these diagnosed diseases (cardiovascular disease, mental disorders, musculoskeletal disorders, respiratory disease or other diseases).

Work ability score, the first item of the work ability index (Tuomi et al. 1998), was measured as the work ability at present compared to the lifetime best on a scale of 0 (extremely bad) to 10 (excellent).

Other covariates included age (51–67 years), gender (men, women), education (basic school, college-level training, academic degree or other training) and occupational class (white collar, blue collar).

### Statistical analysis

Basic characteristics of the participants were analysed and presented first. We found some evidence of difference in exposures by gender in our study. The previous literature also shows men tend to retire later compared to women (Virtanen et al. 2017) and gender difference in retirement preferences was highlighted in a recent study (Pilipiec et al. 2020), therefore, we performed the analysis stratified by gender and also studied the interaction effect of all exposure variables related to quality of work community and gender on the intention to retire.

We used principal component analysis to study the factor structure of the reasons to continue working beyond the lowest pensionable age and the quality of work environment. Principal component analysis was used to reduce the dimension of the exposure variables for better interpretation. The number of the selected factors was based on eigenvalues greater than one and interpretation of scree-plots. Odds ratios (ORs) and their 95% confidence intervals (CIs) were calculated for the associations of the derived factors and intention to retire from the log binomial regression models. Two models were fitted: Model I present the crude association, while Model II was adjusted for age, occupational class, work ability and comorbidity. Final multivariable model includes all variables from Table 1 and presents the estimates for all variables including covariates stratified by gender. The level of statistical significance was considered at 0.05.

**Table 1:** Distribution of the characteristics of the study population stratified by gender

Characteristics	Total *n* = 1,965	Women *n* = 781	Men *n* = 1184	*P* value
Age (mean, SD)	56.29 (3.42)	56.39 (3.48)	56.31 (3.39)	0.261
Education				< 0.001
Basic school	898 (46.19)	317 (41.12)	581 (49.53)	
College-level training	589 (30.30)	281 (36.45)	308 (26.26)	
Academic degree	115 (5.92)	56 (7.26)	59 (5.03)	
Others	342 (17.59)	117 (15.18)	225 (19.18)	
Occupational class				< 0.001
White collar	280 (14.33)	173 (22.29)	107 (9.08)	
Blue collar	1674 (85.67)	603 (77.71)	1071 (90.92)	
Working hours				< 0.001
Regular day work	1317 (67.40)	583 (75.03)	734 (62.36)	
Regular 2-shift work	109 (5.58)	26 (3.35)	83 (7.05)	
Other working hours	528 (27.02)	168 (21.62)	360 (30.59)	
Comorbidity				0.001
0	700 (40.05)	238 (35.05)	462 (43.22)	
1+	1048 (59.95)	441 (64.95)	607 (56.78)	
Work ability (mean, SD)	6.62 (2.19)	6.67 (2.19)	6.59 (2.20)	0.443
Quality of work community				
Equality at work				0.024
Low	656 (33.83)	239 (31.04)	417 (35.67)	
Intermediate	648 (33.42)	253 (32.86)	395 (33.79)	
High	635 (32.75)	278 (36.10)	357 (30.54)	
Supportive work environment				0.014
Low	667 (33.94)	255 (32.65)	412 (34.80)	
Intermediate	635 (32.32)	233 (29.83)	402 (33.95)	
High	663 (33.74)	293 (37.52)	370 (31.25)	
Flexibility at work				0.352
Low	640 (32.57)	246 (31.50)	394 (33.28)	
Intermediate	363 (18.47)	156 (19.97)	207 (17.48)	
High	962 (48.96)	379 (48.53)	583 (49.24)	
Health or other reason				0.017
Low	610 (31.04)	268 (34.31)	342 (28.89)	
Intermediate	696 (35.42)	275 (35.21)	421 (35.56)	
High	659 (33.54)	238 (30.47)	421 (35.56)	
Outcome				
Intention to retire				0.681
No	1286 (67.45)	510 (68.00)	777 (67.10)	
Yes	621 (32.55)	240 (32.00)	381 (32.90)	

We calculated the predictive margins of each derived factor and gender from the final multivariable model to see the predictive power. Predictive margins calculate the mean predictive value of the intention to retire for women and men in all categories of the exposures. We used Omnibus interaction test using -testparm- code in Stata as postestimation command after the log binomial regression analysis. This gives the p value for the interaction effect between two categorical exposure variables (each exposure variables and gender in this case) with respect to the outcome.

All analyses were done in Stata version 16.

## Results

Table 1 presents the baseline characteristics of the studied population by gender. The mean age of the employees was 56.3 years (SD 3.4) with no significant difference between women and men. Significantly more men had basic education, more women had college-level training and academic degree. The majority (86%) of the employees were in blue-collar occupations with more men in blue-collar occupations (91%) than women (78%). More than two-thirds (69%) of the workers were in regular day work, while only 6% were in regular 2-shift work and 27% had other form of working hours. Significantly more women (75%) were in regular day work compared to men (62%). About 60% of the employees had at least one disease, which was significantly higher among women (65%) than men (57%).

Among work-related factors, significant difference was found between women and men in the distribution of equality at work, supportive work environment and health or other reason. Significantly more men reported low equality at work, low supportive work environment and more women were categorized to low health or other reason. The mean work ability score in the total population was 6.6 (SD 2.2), with no significant difference between women and men. Almost one-third (33%) of all employees reported an intention to retire earlier than expected, with no significant difference between women and men in their intention.

The distribution of different categories of work-related factors among those intending to retire early and their associations are presented by gender in Table 2. A large proportion (> 50%) of both women and men reported low equality at work. Among women, in age, occupational class, work ability and comorbidity adjusted model, moderate or poor work community was associated with 2 to 3.1 times higher odds of intention to retire (OR for moderate 2.32, 95% CI 1.40–3.86 and OR for poor 3.14, 95% CI 1.85–5.33) compared to those who reported high equality at work. Intermediate or low flexibility at work was associated with 3.8- and 3.3-times higher odds of intention to retire (OR for intermediate 3.80, 2.29–6.32 and for low 3.26, 1.97–5.40) compared to high. Employees within the low category of health or other reason was associated with lower odds of intention to retire early (0.58, 0.37–0.91) compared to those in the high category.

**Table 2:** Association of quality of work community and intention to retire early among postal service workers stratified by gender

Characteristics	*n* = 646^†^	Women				Men			
		*n* = 240	%^‡^	Model I	Model II	*n* = 381	%^‡^	Model I	Model II
Quality of work community				OR (95% CI)	OR (95% CI)			OR (95% CI)	OR (95% CI)
Equality at work									
High	90	39	14.39	1	1	48	13.83	1	1
Intermediate	209	83	34.02	**3.07 (1.99–4.71)**	**2.32 (1.40–3.86)**	116	29.90	**2.66 (1.83–3.86)**	**1.89 (1.22–2.94)**
Low	340	116	51.33	**6.27 (4.09–9.62)**	**3.14 (1.85–5.33)**	213	51.95	**6.74 (4.69–9.66)**	**3.10 (1.99–4.82)**
Supportive work environment									
High	377	143	40.17	1	1	227	39.89	1	1
Intermediate	108	49	32.24	**0.71 (0.47–1.06)**	0.97 (0.60–1.57)	55	27.09	**0.56 (0.39–0.80)**	**0.64 (0.42–0.97)**
Low	161	48	19.83	**0.37 (0.25–0.54)**	**0.55 (0.34–0.89)**	99	25.65	**0.52 (0.39–0.69)**	0.76 (0.53–1.07)
Flexibility at work									
High	113	42	14.74	1	1	62	17.17	1	1
Intermediate	249	95	42.99	**4.36 (2.86–6.65)**	**3.80 (2.29–6.32)**	145	37.08	**2.84 (2.02–4.00)**	**2.67 (1.75–4.08)**
Low	284	103	42.21	**4.26 (2.79–6.40)**	**3.26 (1.97–5.40)**	174	42.86	**3.62 (2.58–5.06)**	**3.14 (2.07–4.77)**
Health or other reason									
High	256	91	39.91	1	1	158	38.16	1	1
Intermediate	186	65	24.44	**0.49 (0.33–0.72)**	**0.57 (0.36–0.90)**	112	27.45	**0.61 (0.46–0.82)**	**0.55 (0.39–0.79)**
Low	204	84	32.81	0.73 (0.51–1.07)	**0.58 (0.37–0.91)**	111	33.04	0.80 (0.59–1.09)	**0.56 (0.38–0.81)**

Bold values indicate the statistically significant estimates

Model I: crude model, Model II: adjusted for age, occupational class, work ability and comorbidity

^†^Number of people reported the outcome

^‡^Percentage of people reported the outcome per category of the exposure variables

Similar, yet somewhat weaker associations were found for men. Intermediate to low equality at work categories were associated with increased odds of intention to retire early (OR for moderate 1.89, 1.22–2.94 and for poor 3.10, 1.99–4.82). Intermediate to low flexibility at work was associated with higher odds of intention to retire (OR for intermediate 2.67, 1.75–4.08 and for low 3.14, 2.07–4.77). Lower odds of intention to retire was also found for employees with intermediate supportive work environment (0.64, 0.42–0.97) and intermediate to low health or other reason compared to high (OR for moderate 0.55, 0.39–0.79 and for poor 0.56, 0.38–0.81).

Age, education, occupational class, working hours, number of diagnosed diseases and work ability were included in the multivariable model in Table 3, which shows that intermediate to low equality at work was associated with higher likelihood of intention to retire among both women (OR for poor 2.77, 1.60–4.81) and men (OR for poor 2.84, 1.80–3.51). Intermediate or low flexibility at work was also associated with higher odds of intention to retire for both women (OR for low 3.30, 1.94–5.60) and men (OR for low 2.91, 1.88–4.50). Lower likelihood of intention to retire was found due to low supportive work environment for women only (0.52, 0.31–0.81), while intermediate health or other reason was associated among men only (0.65, 0.43–0.98) compared to high.

**Table 3:** Association of quality of work community with the intention to retire early among postal service workers stratified by gender

Characteristics	Women	Men
	OR (95% CI)	OR (95% CI)
Age	0.98 (0.92–1.04)	1.00 (0.95–1.04)
Education		
Basic school	1	1
College-level training	1.19 (0.75–1.88)	0.92 (0.62–1.35)
Academic degree	0.41 (0.15–1.15)	0.44 (0.18–1.08)
Others	0.80 (0.44–1.43)	0.67 (0.44–1.01)
Occupational class		
White collar	1	1
Blue collar	1.39 (0.70–2.75)	1.01 (0.45–2.25)
Working hours		
Regular day work	1	1
Regular 2-shift work	1.51 (0.46–4.98)	0.53 (0.28–1.02)
Other working hours	0.88 (0.53–1.39)	1.01 (0.72–1.44)
Comorbidity		
0	1	1
1+	**1.72 (1.09–2.71)**	**2.49 (1.77–3.51)**
Work ability	**0.71 (0.64–0.79)**	**0.74 (0.68–0.80)**
Quality of work community		
Equality at work		
High	1	1
Intermediate	**2.13 (1.25–3.61)**	**1.91 (1.21–3.00)**
Low	**2.77 (1.60–4.81)**	**2.84 (1.80–4.48)**
Supportive work environment		
High	1	1
Intermediate	1.22 (0.69–2.16)	0.86 (0.54–1.38)
Low	**0.52 (0.31–0.89)**	0.79 (0.54–1.16)
Flexibility at work		
High	1	1
Intermediate	**3.28 (1.87–5.77)**	**2.16 (1.37–3.41)**
Low	**3.30 (1.94–5.60)**	**2.91 (1.88–4.50)**
Health or other reason		
High	1	1
Intermediate	0.77 (0.43–1.36)	**0.65 (0.43–0.98)**
Low	0.68 (0.39–1.20)	0.71 (0.47–1.11)

Bold values indicate the statistically significant estimates

A multivariable model simultaneously adjusted for all studied variables

**Fig. 1:**
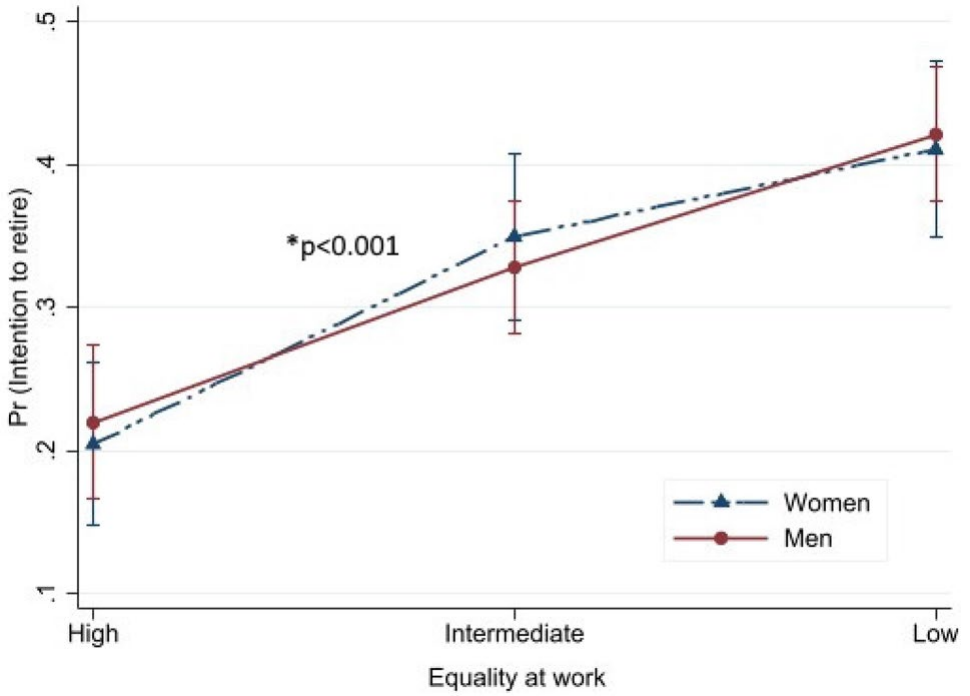
Predicted probability and their 95% CIs of intention to retire early among postal service workers as predicted by equality at separately for women and men (color figure online)

**Fig. 2:**
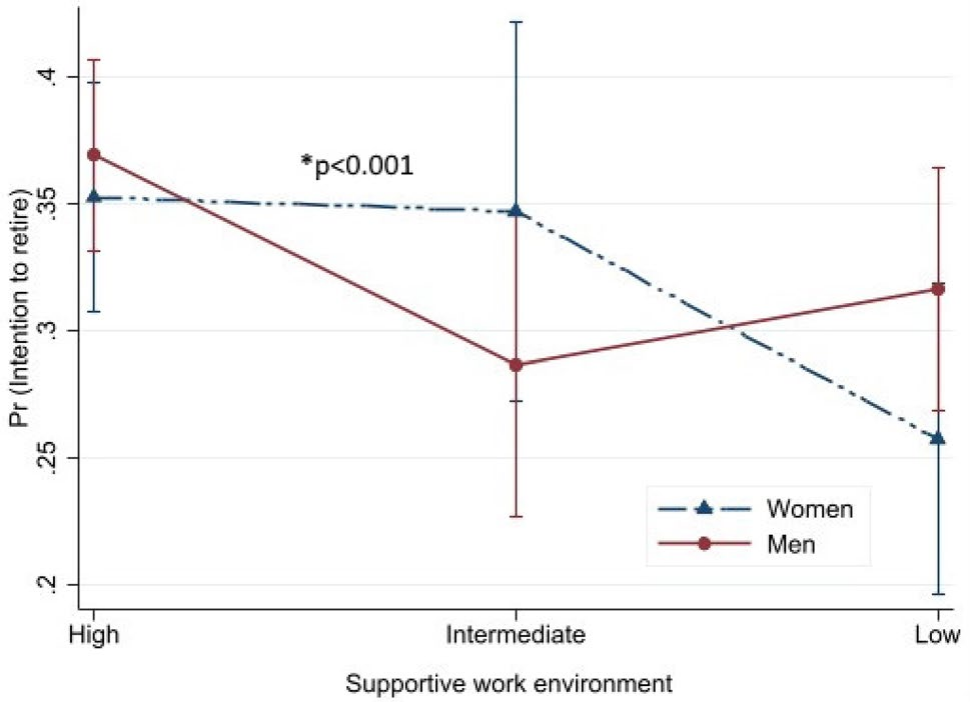
Predicted probability and their 95% CIs of intention to retire early among postal service workers as predicted by supportive work environment separately for women and men (color figure online)

**Fig. 3:**
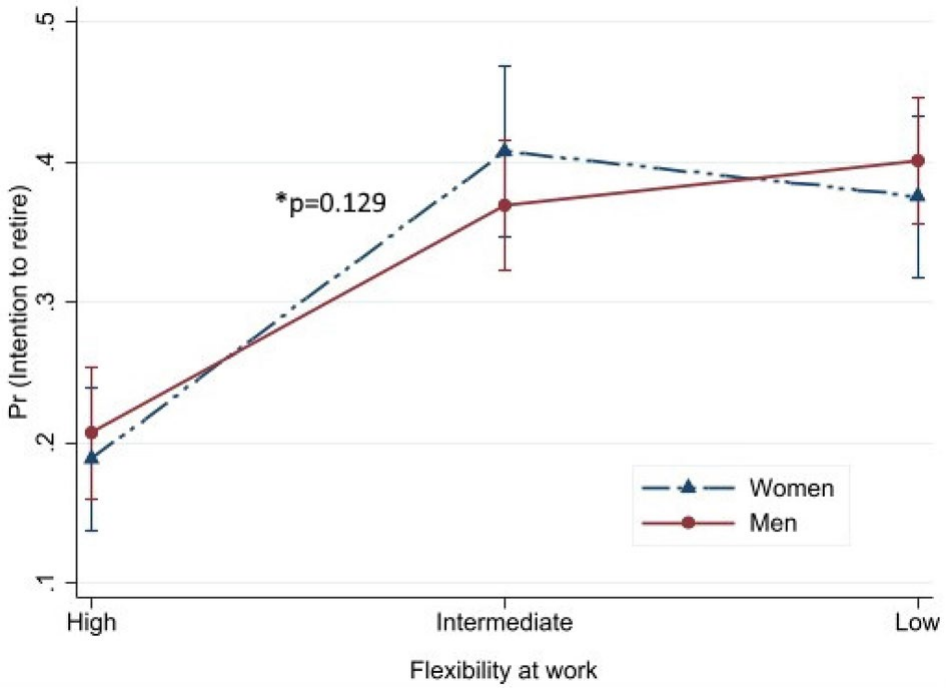
Predicted probability and their 95% CIs of intention to retire early among postal service workers as predicted by flexibility at work separately for women and men (color figure online)

**Fig. 4:**
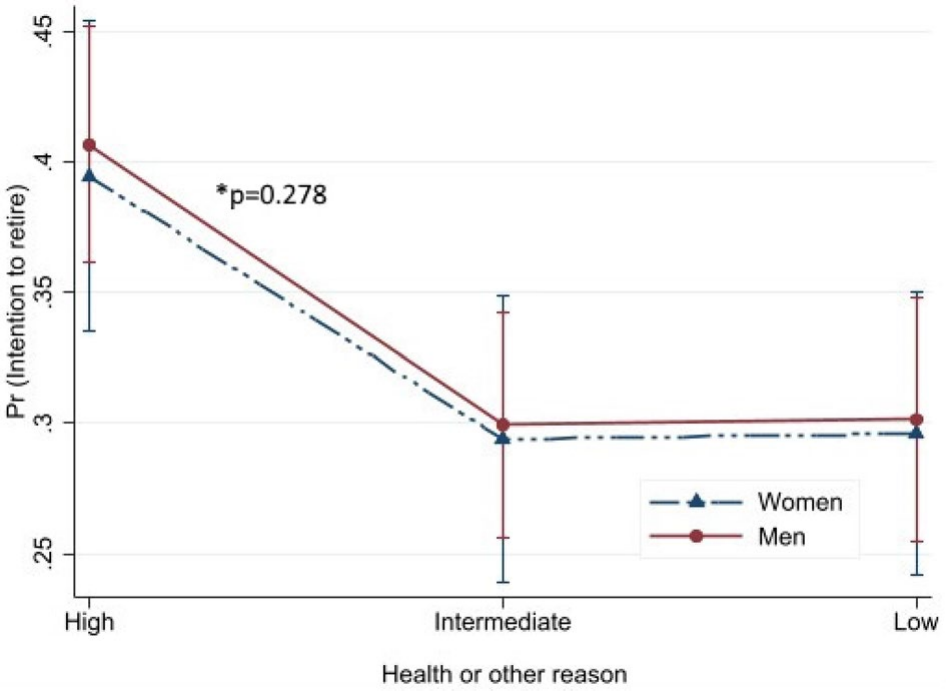
Predicted probability and their 95% CIs of intention to retire early among postal service workers as predicted by health or other reason separately for women and men (color figure online)

Predicted probabilities with their 95% CIs for intention to retire are presented in Figs. 1, 2, 3, 4 to illustrate the interaction effect of the four major exposure variables (equality at work, supportive work environment, flexibility at work and health or other reason) and gender. In general, the effect of exposure differs by gender if the lines are not parallel to each other. Probability of intention to retire increased alongside the level of equality at work from high to low (Fig. 1). This association was different between men and women (*p* for interaction < 0.001). Somewhat decreasing probability of intention to retire was found from high to low supportive work environment (Fig. 2). Also, this association was modified by gender (*p* < 0.001). For women, almost no difference was found for the high and intermediate supportive work environments, while the probability decreased from intermediate to low. Increased predictive probability was found from high to low flexibility at work but the difference between gender was not statistically significant (*p* = 0.129) (Fig. 3). The predictive probability of intention to retire due to health or other reason exposure (Fig. 4) shows no significant effect modification by gender (*p* = 0.278). The lines were parallel to each other for all levels of the exposures.

## Discussion

We found that about one third of the employees intended to retire before statutory retirement age, with no significant gender difference. Quality of work community was associated with intention to retire early both among women and men. Higher likelihood of intention to retire was found among women and men reporting intermediate or low equality at work and intermediate or low flexibility at work, while lower likelihood was found among those reporting low supportive work environment for women and intermediate health or other reason for men. Predicted probability of intention to retire early was higher alongside the perception of high to low equality at work and flexibility at work, while probability was lower alongside the perception of high to low supportive work environment and health or other reason. Statistically significant differences were found between men and women in different levels of equality at work and supportive work environment but not for flexibility at work and health or other reason. Among other factors, those having good work ability were less likely to report intention to retire early and those having a higher number of diagnosed diseases had higher likelihood of intention to retire early among both genders.

Relatively small number of study participants intended to retire before statutory retirement age, which is in line with the current policy reform towards increasing retirement age. In an earlier survey among Finnish working-age people on their intention to continue to work past the lowest pensionable age, 60% of the respondents reported that they would be willing to continue to work, which means 40% intended to retire, for one reason or another (Perkiö-Mäkelä and Hirvonen 2012). An earlier study from Australia reported that about 26% of people working in paid employment intend to retire before their actual retirement age (Taylor et al. 2014). Conversely, a Swedish study reported that a majority of their study participants expected to retire at the official retirement age or earlier (Sousa-Ribeiro et al. 2021).

Retirement has also been studied by looking at which non-health reasons are connected to it, which means factors that push people out of work life (push factors) before the state pension age and pull people towards retirement (pull factors), such as wishes from family and the employee for more time for leisure activities etc. (Andersen et al. 2020). Stay factors, on the other hand, refer to the factors that encourage older workers to voluntarily prolong their working, such as fulfilling work or appreciation of the work community (Andersen et al. 2020). Our study further investigated how the different factors are connected and how they relate to the intention to retire.

PCA showed that those who appreciated flexibility of work and working hours were the least likely to choose financial reasons as the main reasons to keep working beyond the lowest pension age. Our study investigated the role of the psychosocial work environment in terms of equality at work, flexibility at work, supportive work environment and health or other reasons. Low equality at work as well as low flexibility at work was a strong predictor of intention to retire among both women and men. The association was particularly strong for women. In our study the equality of the work community comprised of experiencing equality such as feeling of appreciation, confidence towards the employer, commitment to the work, motivation and feeling of being treated fairly. The positive values of these factors separately may be a good motivation for any workers to stay at work at least until official retirement age. Only 14% of the workers who had high equality at work had intention to retire before their minimal pensionable age in this study compared to the 51% among those perceiving a low equality at work. A systematic review reported strong evidence for the association of greater resources and job satisfaction as a measure of psychosocial factors at work with later retirement (Browne et al. 2019).

We also found that having heavy work tasks and low flexibility at work were associated with the intention to retire before pensionable age. Our findings are supported by an earlier study from the Netherlands which reports that push, pull and jump factors are strongly associated with retirement intentions of older workers (Andersen et al. 2020). Workers on the edge of being pushed out due to strenuous physical work could stay at work longer if their work is made less strenuous or more flexible which means that, suppressing push factors may prolong work career of the workers. Similar to our findings, the financial opportunity to early retirement played a major role in the actualization of retirement intentions (Reeuwijk et al. 2013).

It is plausible that those under financial pressure are more likely to perceive retirement as unaffordable (McManus et al. 2007). In fact, the most reported reason for returning to the workforce after initial retirement is “financial need” (42%) among Australian older workers (Australian Bureau of Statistics. 2017). This shows that factors such as poor health, caregiving and work conditions may have a limited effect on employment decisions for workers who are under financial pressure. They simply have little choice but to remain at work (Garcia et al. 2014).

We found that low supportive work environment and health or other reasons were associated with lesser likelihood of intention to retire before the statutory retirement age. The supportive work environment in our study comprised of good and functional working environment and interesting and challenging work tasks. This shows that work community at the individual level is more important for continuation of job rather than the organizational level factors. Our results contradict earlier findings which reported that organizational changes, conflicts at work, significant work pressures and lacking opportunities to utilize one’s skills and knowledge at work are all found to be factors that push people towards retirement (Reeuwijk et al. 2013). In addition, fair management of the organization has been found to be an important factor in extending work careers, while staff reductions increased the desire to retire before age 65 (Peutere et al. 2017).

Having one or more disease was associated with intention to retire early. This finding is in line with earlier findings which shows that poor physical health is an expected reason for retiring (Meng et al. 2020; Wahrendorf et al. 2017). Another recent study from Sweden reported that people with better health and positive work prospects were less likely to support retiring earlier (Sousa-Ribeiro et al. 2021). Having good work ability was associated with a lesser likelihood to retire early in our study. Good work ability has been reported to be associated with decreased early retirement intentions in an earlier study (von Bonsdorff 2010). Another cross-sectional study from Denmark found that work ability in both genders was associated with retirement ideation; poor work ability was found to be a major push factor (Thorsen et al. 2012). We found no age difference in retirement intention in our study of people beyond 50 years of age. However, being older was associated with decreased probability of early retirement intentions in men in a Danish study (Thorsen et al. 2012).

Physically demanding work increased early retirement, while good health best supported continuing to work ideation (Perkiö-Mäkelä and Hirvonen 2012). For those employed in physically light work, financial reasons, meaningful and challenging work and flexible work hours were the primary reasons to potentially continue working past the lowest pensionable age when occupational groups were compared according to workload (low, medium, high physical workload) (Perkiö-Mäkelä and Hirvonen 2012). In our study, physical work environment related factors were not directly studied, but in terms of occupational task we found no significant difference in intention to retire early between blue- and white-collar workers. Postal service workers are typically working partly in office while sorting the mail and then they deliver it by foot, bicycle or car. In another study, occupational status has been found to be associated with continuing to work past the lowest pensionable age (Virtanen et al. 2017). Employees in higher occupational positions are twice as likely to continue to work than those in lower occupational positions (Virtanen et al. 2017). This result was explained by the lighter physical workload, better autonomy regarding work time, and better perceived work ability, of those employees in higher positions (Virtanen et al. 2017).

Although work-related factors have been studied extensively, we found no earlier studies focusing on the role of the quality of work community on retirement intentions of postal workers, and future studies should explore the longitudinal association of these psychosocial and work organizational factors for the stronger evidence.

One of the major strengths of this study is a large and homogenous sample of older workers. The nature of postal service work is similar in many countries; therefore, the findings may be generalizable beyond the study population of the current study. However, there are methodological issues that should be considered while interpreting the results. The causal inference between the exposure and the outcome cannot be established because of the cross-sectional nature of the study design. The use of self-reported responses for exposures is a source of uncontrolled measurement error due to common method variance (Podsakoff et al. 2003). The observed association could have been overestimated as the exposure and outcome variables were measured at the same time. Nevertheless, we adjusted the regression models for potential confounders which reduced the likelihood of overestimation. We additionally ran the exposure–gender interaction analysis and presented predictive margins to show the gender difference in the outcome at different levels of the exposures. A longitudinal design would provide an additional benefit, particularly if participants were followed into their retirement to explore the relevant work characteristics that influenced their retirement.

In conclusion, one third of the older post service workers had intention to retire before their statutory retirement age. The intention to retire was strongly associated with quality of work community among both women and men. This suggests that a good working environment consisting of flexible work time, treating workers fairly and appreciation may help workers’ motivation to work longer.
